# BDNF/TrkB axis activation promotes epithelial–mesenchymal transition in idiopathic pulmonary fibrosis

**DOI:** 10.1186/s12967-017-1298-1

**Published:** 2017-09-22

**Authors:** Emanuela Cherubini, Salvatore Mariotta, Davide Scozzi, Rita Mancini, Giorgia Osman, Michela D’Ascanio, Pierdonato Bruno, Giuseppe Cardillo, Alberto Ricci

**Affiliations:** 1 Department of Clinical and Molecular Medicine, Sapienza University of Rome, Rome, Italy; 2 Thoracic Surgery Unit, Ospedale Carlo Forlanini, Azienda Ospedaliera San Camillo Forlanini, Rome, Italy

**Keywords:** BDNF, TrkB, EMT, Idiopathic pulmonary fibrosis, Lung

## Abstract

**Background:**

Neurotrophins (NT) belongs to a family of growth factors which promotes neurons survival and differentiation. Increasing evidence show that NT and their receptor are expressed in lung tissues suggesting a possible role in lung health and disease. Here we investigated the expression and functional role of the TrkB/BDNF axis in idiopathic pulmonary fibrotic lung (myo)fibroblasts.

**Methods:**

Lung fibroblast were isolated from IPF patients and characterized for the expression of mesenchymal markers in comparison to normal lung fibroblasts isolated from non-IPF controls.

**Results:**

BDNF treatment promoted mesenchymal differentiation and this effect was counteracted by the TrkB inhibitor K252a. In this regard, we showed that K252a treatment was able to control the expression of transcription factors involved in epithelial to mesenchymal transition (EMT). Accordingly, K252a treatment reduced matrix metalloproteinase-9 enzyme activity and E-cadherin expression while increased cytoplasmic β-catenin expression.

**Conclusions:**

Our results suggest that BDNF/TrkB axis plays a role in EMT promoting the acquisition of (myo)fibroblast cell phenotype in IPF. Targeting BDNF/TrkB seems to represent a viable approach in order to prevent EMT dependent lung fibrosis.

## Background

Idiopathic pulmonary fibrosis (IPF) is a progressive lung disease characterized by fibroblast accumulation, collagen deposition, and parenchyma destruction [1, 2]. While the exact cause of IPF is unknown, an aberrant activation of the alveolar epithelial cell (AEC) is widely recognized a key feature associated to IPF pathogenesis [3]. According to the current understanding, recurrent AEC injuries initiate a cascade which leads to aberrant collagen deposition and tracheobronchial distortion [1, 2]. Despite (myo)fibroblasts seem to play a critical role in this process, their origin within the lung is still controversial. Using a mouse model of bleomycin induced pulmonary fibrosis in BM GFP^+^ chimera mice, Hashimoto et al. demonstrated that (myo)fibroblasts can directly differentiate from circulating bone marrow derived cells homing to the lung [4]. In addition, (myo)fibroblasts have also been proposed to directly differentiate from TGF-β activated resting lung fibroblasts [5]. However, original findings on the role of epithelial-to-mesenchymal transition (EMT) in pulmonary fibrosis are recently providing new insight into the mechanisms of myofibroblast differentiation [6]. EMT is defined as a process by which epithelial cells lose the cell polarity and cell–cell adhesion, and gain migratory and invasive properties to become mesenchymal stem cells [7]. Disruption of cell contacts [7], hypoxia and inflammation [8] as well as by oxidative stress [9] are commonly recognized as major triggers for EMT.

EMT is a molecular process by which epithelial cells trans-differentiate into motile mesenchymal cells. It contributes pathologically to fibrosis and cancer progression [10]. This phenomenon is mediated by specific transcription molecules like Twist and Snail (1 and 2-Slug-) that activate the EMT during the development of fibrosis and cancer [11].

Neurotrophins (NTs), nerve growth factor (NGF), brain derived neurotrophic factor (BDNF) neurotrophin 3 (NT3) and NT4, are a family of highly conserved polypeptide growth factors involved in different cellular functions [12]. Besides their role in survival, differentiation, and function of peripheral nerves and brain neurons of mammals [12, 13], NTs exert additional activities in tumor biology [14, 15] and chronic inflammation [16–18]. Moreover, previous findings from our group support a possible role for NT in lung fibrosis [17]. NTs exert their effects binding two structurally unrelated receptors: the p75 low affinity receptor, a member of the tumor necrosis factor receptor superfamily, and the high affinity tyrosine kinase receptors (Trks). While p75 displays the capability of binding to all NTs, the Trk receptors exhibit ligand selectivity. In particular, TrkB mostly exhibits BDNF and NT4 binding specificity [12].

BDNF-dependent TrkB activation has been recently associated to EMT-like transformation through the Twist-Snail signaling axis, which is dependent on the MAPK pathway [19–21]. However, most of these findings are based on model of malignant tumor cells in which the TrKB–BDNF axis has been demonstrated to be dysregulated. Whether this mechanism is specific for malignant transformation it is currently not clear. IPF and cancer possess many similarities that suggest the vision of IPF as a cancer-like disease. Similar signaling cascades, oncogene activation, epigenetic and genetic alterations have been reported [22].

Here we aimed to evaluate the potential significance of the TrkB–BDNF axis on EMT-dependent (myo)fibroblasts differentiation in IPF.

## Methods

### Human pulmonary fibroblasts cell cultures

Human lung fibroblasts were grown from lung biopsies following enzymatic disaggregation by collagenase and protease [17]. Lung tissue from patients with IPF (n = 10) (mean age ± SD 61.5 ± 5.3; male 6; female 4) who underwent lung biopsies was collected and used to generate fibroblast cell cultures. The diagnosis of IPF was confirmed based on clinical, radiological and isto-pathological findings. Tissue isolated from five donor lungs (surgical treatment of pulmonary benign nodules) (mean age 72 + 4.4; 3 male; 2 female) was used to generate normal fibroblasts cell cultures.

From these surgical specimens only five primary fibroblast cell cultures from IPF and two from normal lung, that displayed good viability and suitable for all the subsequent experiments, was obtained.

Cells were grown in RPMI medium supplemented with 10% FBS, 100 units/mL penicillin, 100 µg/mL streptomycin and 4 mM l-glutamine (LONZA). The medium was replaced twice a week. The cells were incubated at 37 °C in a humidified atmosphere containing 20% O_2_ and 5% CO_2_. Fibroblasts were characterized by flow cytometry and immunocytochemistry. The pellet obtained from the cultures were used for protein and RNA extraction. The cultures were used to study the functional effects of BDNF on BDNF/TrkB axis. To better clarify BDNF effects the serine/threonine protein kinase inhibitor (K252a) was used. K252a is considered potent inhibitor of all neurotrophin tyrosine protein kinase activity [23].

All the patients signed an informed consent.

### Western blot analysis

Proteins extracted from fibroblast homogenates were separated by 10% SDS–polyacrylamide gel electrophoresis and then transferred to a 0.45 μm pure nitrocellulose membrane (Bio-Rad) by transfer buffer. The blots were blocked with milk at 5% for 1 h and incubated overnight at 4 °C with the monoclonal antibodies: anti-Human N-cadherin (1 mg/mL) (R&D system), anti-vimentin cloneV9 (1:250) (SIGMA), anti-a-actin (N-19) (1:200) (Santa Cruz), anti E-cadherin (G-10) (Santa Cruz) and anti-cytokeratin, pan (mixture) C2562 (SIGMA), anti-β-catenin (E-5) (Santa Cruz) and anti-MMP-9 (626–644) (Ab-3) (Calbiochem). Primary antibodies-bound membranes were incubated at 4 °C overnight with HRP-conjugated secondary antibody (1:4000) anti-mouse (SIGMA). Further details are reported in a previous study [19]. For detection, an ECL chemiluminescence system (Therma Scientific) was used.

### Immunofluorescence

For immunofluorescence assay, cells were seeded onto multi-chamber slides (Becton–Dickinson) at a density of 1  ×  10^4^ cells. After 5 days of treatment, the slides were washed in phosphate-buffered saline (PBS) with calcium and magnesium, fixed in paraformaldehyde 4% for 15 min and permeabilized in Tris buffered saline (TBS) 0.05 M pH 7.4 with 1% Triton X-100 for 15 min. After blocking in TBS 10% serum for 30 min, slides were incubated with the monoclonal antibodies: anti-Human N-cadherin (1 mg/mL) (R&D system AF 6426), anti-vimentin cloneV9 (1:250) (SIGMA), anti-a-actin (N-19) (1:200) (Santa Cruz), anti E-cadherin (G-10) (Santa Cruz) and anti-cytokeratin, pan (mixture) C2562 (SIGMA), for 1 h at 4 °C. After washing in TBS, they were incubated with goat anti-rabbit Alexa Fluor 488 and goat anti-mouse Alexa Fluor 594 antibodies (invitrogen) for 30 min and finally washed twice in TBS. Nuclei were counterstained with Hoechst 33342 (Sigma) 1 μg/mL for 10 min. Images were acquired with an Olympus BX51 fluorescence microscope and analyzed with I.A.S. software (Delta Sistemi, Legnano, Italy). The brightness and contrast of the acquired images were adjusted, and the figures were generated using Adobe Photoshop 7.0. Cells not treated with RA were considered to be negative controls.

### Immuno-fluorescent method for flow cytometry

Flow cytometry was used in order to evaluate the expression of cell surface markers on cultured fibroblast using a FACS Calibur cyto-fluorimeter (Becton–Dickinson, Sunnyvale, CA, USA) (FCM). Briefly, cells were treated with the BDNF (10 ng/mL) (SIGMA) and TrKB inhibitor K252a (3 nM) (SIGMA) for 10 days. After the treatment cells were stained with specific primary antibody followed by a secondary FITC-conjugated antibody before to be analyzed by FCM.

### RNA extraction and semi-quantitative RT-PCR analysis

RNA extraction and cDNA synthesis were performed as previously described [24]. The 18S RNA was used to normalize the amount of total RNA present in each reaction. Specific sequences targeted against Snail gene for 5′gcgcagctctaatccaga-3′ and rev5′-atctccggaggtgggatg-3′; Twist gene for 5′-agctacgccttctggtct-3′ and rev 5′-ccttctctggaacaatgacatc-3′ and Zeb gene for: 5′-gggaggagcagtgaaagaga-3′, Rev: 5-tttcttgcccttcctttctg-3′, were obtained from Sigma Aldrich. PCR products were visualized on a 2% agarose gel after ethidium bromide staining. Three independent experiments were performed for each sample. The intensities of the bands on gels were measured by densitometry, using the ImageJ software (version 1.32j).

### Zymography

Proteins were separated by electrophoresis under denaturing (SDS), non-reducing conditions, with a 10% polyacrylamide gel containing 1–2 mg/mL gelatin. After electrophoresis, the gel was washed and incubated overnight in an appropriate activation buffer at 37 °C. The gel was then stained with Coomassie^®^ Brilliant Blue R250 (Kodak) in 30% methanol, 7.5% acetic acid and washed with water at 22 °C for 20 min. The MMPs were detected as clear bands against a blue background of un-degraded substrate. The clear bands in the gel were quantified by densitometry.

## Trypan blue exclusion test of cell viability

The Trypan blue exclusion test was used to determined viable cell number in cell suspension. Damaged or not viable cell incorporated the trypan blue dye and assumed a blue staining.

IPF fibroblast cell line were seeded in 60 mm Petri dishes at density of 2 × 105 cells in RPMI medium supplemented with 5% FBS. RPMI1640 medium with BDNF (10 nM) with or without k252a inhibitor were added in the cell separately, as described above. Effects of treatment on cell migration the IPF fibroblast cell line were evaluated after 24, 48 and 120 h. The number of viable cells present in a cell suspension was obtained by trypan-blue assay (Sigma Aldrich). Briefly, 10 mL of cells was aseptically transferred to a 0.2 mL clear Eppendorf tube and incubated for 2 min at room temperature with a equal volume of trypan blue solution. The cell number was determined by counting the viable cells in a hemocytometer. The percentage of viable cells from each tube after incubation with material extracts was obtained by applying the following equation: $\%\,{\text{viable cells}} = \left( {{\text{VC}}/{\text{TC}}} \right) \times 100,$ where VC = viable cells counted and TC = total cells counted (stained plus unstained cells). The experiments were performed in triplicate.

### Wound healing assay

Fibroblast from IPF lungs (UIP/IPF) were seeded in 60 mm Petri dishes at density of 5 × 10^5^ cells. After 48 h, the cells were confluent and at a concentration of 1 × 10^6^ cells/mL and cultured in medium containing 10% FBS to nearly confluent cell monolayers. The cell were then starved overnight in serum free RPMI-1640 medium. A linear wound was generated in the monolayer with a sterile 100 μL plastic pipette tip. Any cellular debris was removed by washing the coverslips with phosphate buffer saline (PBS). RPMI1640 medium with BDNF (10 nM) or k252a inhibitor were added in the cell separately, cell lines cultured with RPMI1640 alone were considered as controls (C). Effects of treatment on cell migration were evaluated for 120 min. Three representative images from each point of the scratched areas under each condition were photographed to estimate the relative migration cells. Migration ability was assessed by measuring changes in the size of the wounded areas. All the experiments were performed in triplicate.

### Statistical analysis

Data are expressed as mean ± SD. Statistical significance was assessed using unpaired Student t test. Multiple groups were analyzed by the one-way ANOVA tests. Statistical analysis was performed by using the SPSS software (IBM SPSS Statistics US) and a p value < 0.05 was considered significant.

## Results

### Lung fibroblast from human IPF show higher levels of EMT

Human primary cell lines of fibroblast derived from either normal and IPF lungs were assessed for EMT markers using flow cytometry.

N-cadherin, vimentin and α-actin were used as mesenchymal cell markers, whereas E-cadherin and pan-cytokeratin were used as epithelial cell markers.

**Fig. 1:**
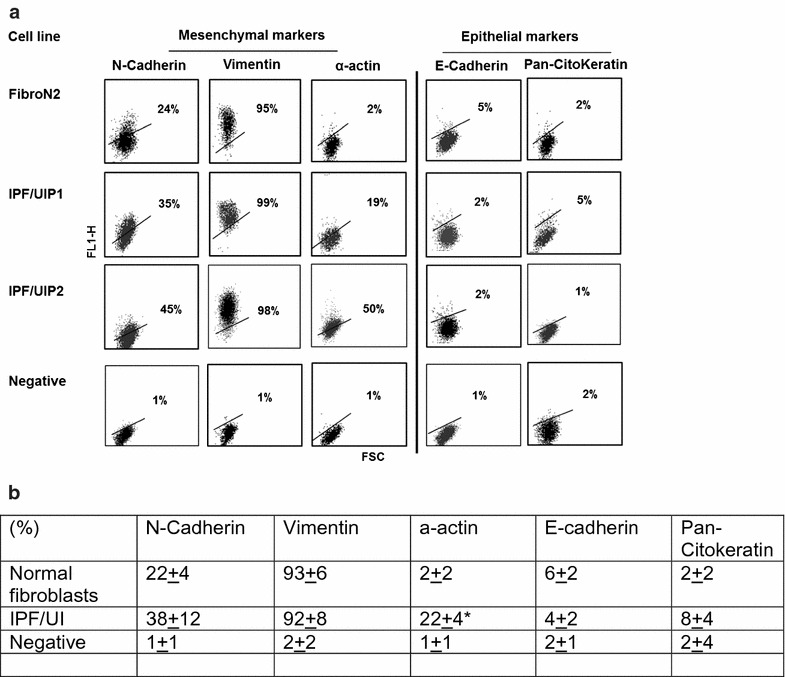
Analysis of mesenchymal and epithelial markers in representative idiopathic pulmonary fibrosis (IPF/UIP1 and 2) and normal fibroblast (FibroN2) cell cultures using flow cytometry. **a** Analysis of mesenchymal and epithelial markers in representative idiopathic pulmonary fibrosis (IPF/UIP1 and 2) and normal fibroblast (FibroN2) cell cultures using flow cytometry. **b** Panel B represents the mean values ± SD of the results obtained in the two normal fibroblast cell cultures considered as controls in comparison with the five fibroblast cell cultures obtained from IPF patients. * p < 0.01 versus normal fibroblast cell cultures. Negative sample was generated with the corresponding isotope (negative)

We observed that approximately 35% of our cultured fibroblasts derived from IPF lung expressed N-cadherin where almost all of them 99% were positive for vimentin. In comparison, only 24% of the normal control fibroblast express N-cadherin and not more than 95% were positive for vimentin (Fig. 1).

Regarding α-actin we found its expression in almost (19–50%) of the IPF derived fibroblast compared to the 2% positive cells observed in normal control fibroblasts cell lines (Fig. 1).

These results indicate that fibroblast cultures obtained from IPF lungs are more enriched with cells expressing EMT markers compared to normal fibroblast cell lines. In addition, the percentage of cells expressing epithelial markers such as E-cadherin and pan-cytokeratin was extremely low in both condition (< 5%) confirming the absence of epithelial cell contamination in our fibroblast cell cultures (Fig. 1).

### BDNF/Trk axis promotes EMT associated cadherin switching in lung fibroblast

**Fig. 2:**
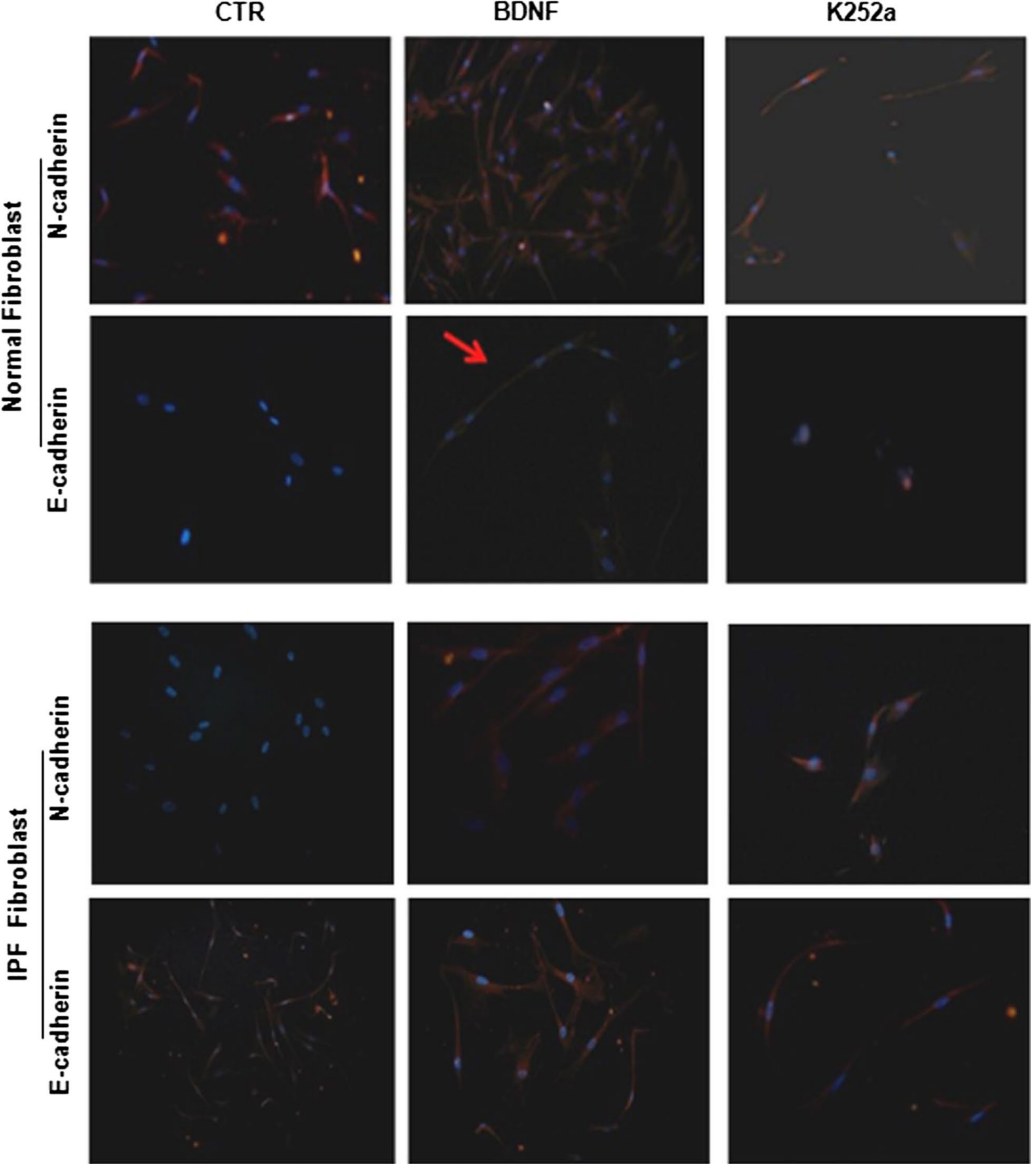
K252a by BDNF/TrkB axis modulate the mesenchymal and epithelial markers in fibroblast cell line. Representative immunofluorescence pictures showing that BDNF binding to TrKB receptor up-regulates the expression of mesenchymal markers. These effects are inhibited by the specific TrkB receptor selective inhibitor K252a

**Fig. 3:**
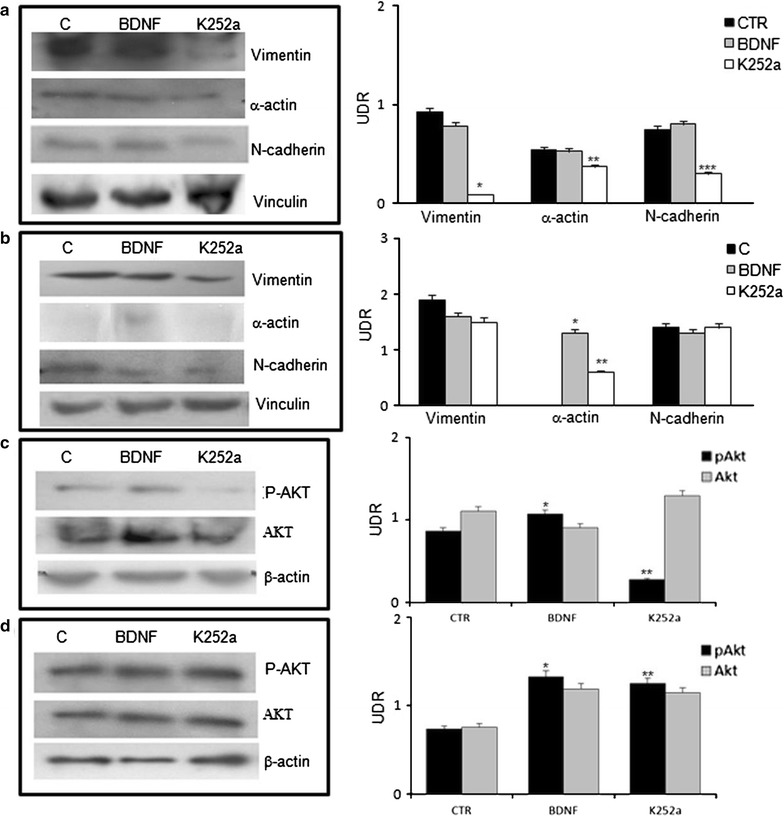
Cell signaling in idiopathic pulmonary fibrosis. Western blot analysis of the expression of mesenchymal marchers in homogenates of fibroblast cell cultures obtained from normal and IPF subjects. As noticeable, the expression of mesenchymal marker protein was decreased in the presence of the TrkB receptor blocker K252a in IPF fibroblast homogenates (*p < 0.001; **p < 0.05; ^***^p < 0,01 vs BDNF treated and controls) (**a**). Contrarily, the presence of the BDNF increased α-actin expression in normal fibroblast (*p < 0.001; **p < 0.01 vs controls) (**b**). The addition of TrkB receptor selective inhibitor K252a reduces the phosphorylation of AKT in IPF fibroblasts (*p < 0.05; **p < 0.001 vs K252a treated cells) (**c**). This effect was slightly noticeable in normal fibroblasts (*p < 0.01; **p < 0.05 vs K252a treated cells) (**d**). Results were statistically evaluated by using Student’s t test

Previous work from our group show that TrKB and BDNF are highly expressed on human IPF lung fibroblast [17]. Here, we investigate the effects of TrKB stimulation and inhibition in terms of fibroblast mesenchymal differentiation. Using fluorescence microscopy we showed that the addition of BDNF (10 ng/mL) to culture medium promote the increase of N-cadherin expression in human lung fibroblast (Fig. 2). This effect was more pronounced in fibroblasts derived from IPF lung compared with the normal control (Fig. 3). This phenomenon may be due to the different phenotype and activity of IPF fibroblasts in comparison to normal ones. The N-cadherin expression was associated with TrKB activation since the treatment with the TrK inhibitor K252a (3 µM) attenuated this effect (Fig. 2 and 3).

#### Effect of K252a inhibitor on the BDNF/TrKB axis

**Fig. 4:**
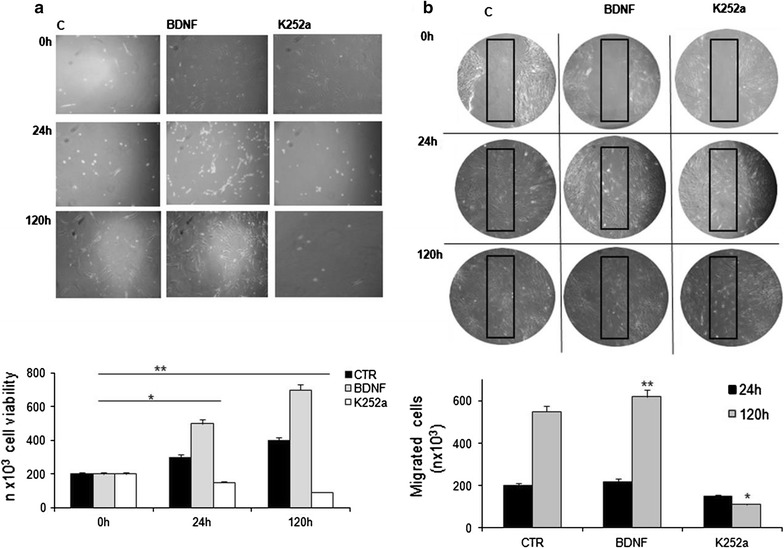
Effect of the TrkB receptor blocker K252a on fibroblast cell growth. **a** K252a induced a decrease in cell growth in comparison to cell line treated with BDNF and no treated cells after 120 h (C). The number of viable cells present in a cell suspension was obtained by tryplan-blue staining. Histogram at the bottom represents the number of fibroblast cell viability obtained in the Trypan blue exclusion test after 24 h of treatment with indirubin. The asterisk indicates significant difference compared to the BDNF treated cell and control group (*p < 0.05, ** p < 0.01, ANOVA followed by Tukey’s test). Each bar represents the mean ± SD of three independent experiments. **b** Migration ability of fibroblast cell lines determined by wound healing assay. Cell lines treated with BDNF have a higher motility into the wounded area, in comparison those treated with TrkB inhibitor K252a (black rectangles). Results were statistically evaluated by Students *t* test (*p < 0.05 K252a treated cells vs controls, **p < 0.01 BDNF treated cells vs controls). The amount of migrated cells was measured by ImageJ software (version 1.32j)

In order to assess the functional effect of BDNF and its K252a dependent inhibition on human IPF fibroblast cell cultures we assessed cell vitality by trypan-blue exclusion cell count and wound healing assay. We observed that the addition of K252a to the medium, inhibited cell proliferation of about 70% in a time dependent way, in comparison with cell lines treated with BDNF (Fig. 4a). Moreover by wound healing assay, we demonstrated that migration of cell, into the wounded area, was reduced by K252a (Fig. 4b).

**Fig. 5:**
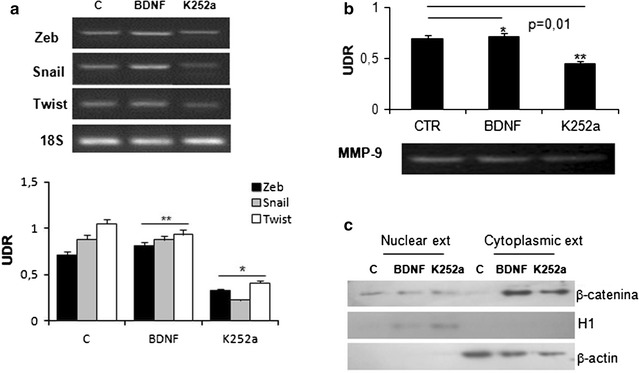
BDNF/TrKB axis activation on transcriptional factors and ECM markers. **a** mRNA levels of Zeb, Snail and Twist changes in the presence of BDNF or TrkB receptor blocker K252a. Note the decrease in the mRNA expression in comparison to BDNF treated cell lines. **b** Zymography assay. Enzyme activity of MPP9 was reduced after k252a treatment. **c** BDNF effects on cytoplasmic and nuclear expression of the β-catenin protein. Increase of the β-catenin cytoplasmic expression induced by BDNF was inhibited blocking TrKB receptor by K252a (*p = 0.003). The nuclear expression of β-catenin was increased in the presence of BDNF. K252a reverted this effect (**p = 0.0007). UDR: densitometric relative unit

In addition, we assessed the mRNA levels of Zeb, Snail and Twist in IPF cell line by PCR. The addition of BDNF (10 nM) to culture medium is able to significantly increase mRNA expression of Zeb, Snail and Twist in comparison to cell line treated with K252a inhibitor. On the contrary, K252a induced a decrease in mRNA expression of same genes in comparison to not treated cells (Fig. 5a).

We have also assessed the matrix metalloproteinase-9 (MPP-9) and β-catenin protein expression. The results, obtained by zymography, showed that the enzyme activity of MPP-9 was reduced after k252a treatment in comparison to BDNF and no treated cell lines (Fig. 5b). Moreover BDNF induced an increased cytoplasmic expression of the β-catenin protein that was not induced K252a. The nuclear expression of β-catenin was not affected by the presence of BDNF or K525a (Fig. 5c).

## Discussion

Increased expression of mesenchymal and reciprocal low expression of epithelial markers induces profound alteration in epithelial cell polarity and morphology, resulting into epithelial-to-mesenchymal transition (EMT) [25, 26]. The EMT has been considered to be critical biological process in epithelial tumor invasion, progression and metastasis. Recent data also implicates TrkB as a regulator of EMT [19, 27] and a direct role in the tumor progression [14, 28–31]. More recently, the high affinity TrkB receptor activation, induced by its specific ligand BDNF, has been demonstrated to induce an EMT-like transformation in epithelial cells through a Twist-Snail signaling axis, which is dependent on the MAPK pathway [21]. Moreover, the inverse correlation between TrkB and E-cadherin expression has been strictly linked to more aggressive phenotype in non small cell lung cancer [19].

The TrkB receptor has been reported to possess an essential role in airway branching and alveolarization [32], and NTs and their receptors have also been considered as intriguing molecules in several adult lung diseases including IPF [33–36]. However, apart from these limited results there is no clear data about the biological role the BDNF/TrkB signaling pathway in idiopathic pulmonary fibrosis.

Our results, for the first time, demonstrate that the activation of BDNF/TrkB signaling pathway plays an important role in EMT in primary fibroblast cell cultures from fibrotic lung and that TrkB may address fibroblasts phenotype.

These data provide new insight that fibroblasts can also form locally by EMT during lung fibrosis. Therefore, a part of fibroblasts located in fibrotic lung may derive from epithelial cells. Epithelium, with the damage of basal membrane, during the fibrotic process within the lung, detach from their functional units losing their morphogenic features. It is well known that type II alveolar epithelial cells possess proliferative potential [37]. In vivo and in vivo results underline that these cells may be progenitors of mesenchymal cells contributing to the pool of expanded fibroblasts during lung injury in mice [37]. Local secretion of pro EMT cytokines, usually found in the transitional environment, may drive these effects. BDNF may have a role in this transition, by binding high affinity TrkB receptor. BDNF is able to induce an increase in mesenchymal markers, but its inhibition, by specific receptor antagonists, promote epithelial marker re-expression [19, 30, 38].

The expression of both mesenchymal and epithelial markers also observed in our results are in line with the observation of different authors that described their co-localization, in the fibrotic lung [25, 37, 39].

Several line of evidences suggest that IPF fibroblasts are able to respond to different inflammatory and non-inflammatory stimuli like several cytokines. Interestingly, BDNF was able to activate Jak/Stat pathway that was also activated by the pro-fibrogenetic and mitogenic IL6 [20, 34, 40, 41]. Therefore, BDNF represents an attractive molecule able to participate to the complex pro-fibro-genetic cytokine network that participate to lung fibrosis in IPF.

These data are in line with the hypothesis that EMT may be part of mechanisms involved in fibroblast and (myo)fibroblast accumulation within the lung in idiopathic pulmonary fibrosis. In experimental models, EMT have been considered as a marker of epithelial dysfunction and damage in response to inflammatory stimuli but may be also considered as a mechanism by which (myo)fibroblasts can be generated, underlining different sources and phenotype of fibroblasts during fibrosis. EMT, in this context, could participate in an aberrant epithelial–mesenchymal communication, through which the activated alveolar cells may release factors that promote and maintain fibro-genesis and allow fibroblasts to survive. Thus fibrosis is a complex phenomenon shared by failing organs, EMT may be considered a pathophysiological dysfunction that lead to epithelial unit loss, and conversion of collagen synthesis. It could not be excluded that different differentiated cells within the lung (endothelium, or myocytes) could also transition to fibroblasts when necessitated, participating to lung remodeling.

## Conclusions

To summarize, the TrkB pathway represents a complex signaling network that is of interest to understand the pathophysiology of different proliferative, inflammatory and degenerative lung disorders and to the potential design of novel therapeutic treatments and combination therapies.

## Abbreviations

EMT: epithelial to mesenchymal transition
BDNF: brain derived neurotrophic factor
IPF: idiopathic pulmonary fibrosis
NTs: neurotrophins
